# Electrical Stimulation Elicits Neural Stem Cells Activation: New Perspectives in CNS Repair

**DOI:** 10.3389/fnhum.2015.00586

**Published:** 2015-10-19

**Authors:** Yanhua Huang, YeE Li, Jian Chen, Hongxing Zhou, Sheng Tan

**Affiliations:** ^1^Department of Neurology, Zhujiang Hospital of Southern Medical University, Guangzhou, China; ^2^Department of Neurology, Dalang Hospital, Dongguan, China

**Keywords:** electrical stimulation, neural stem cells, activation, central nervous system, neural regeneration, repair

## Abstract

Researchers are enthusiastically concerned about neural stem cell (NSC) therapy in a wide array of diseases, including stroke, neurodegenerative disease, spinal cord injury, and depression. Although enormous evidences have demonstrated that neurobehavioral improvement may benefit from NSC-supporting regeneration in animal models, approaches to endogenous and transplanted NSCs are blocked by hurdles of migration, proliferation, maturation, and integration of NSCs. Electrical stimulation (ES) may be a selective non-drug approach for mobilizing NSCs in the central nervous system. This technique is suitable for clinical application, because it is well established and its potential complications are manageable. Here, we provide a comprehensive review of the emerging positive role of different electrical cues in regulating NSC biology *in vitro* and *in vivo*, as well as biomaterial-based and chemical stimulation of NSCs. In the future, ES combined with stem cell therapy or other cues probably becomes an approach for promoting brain repair.

## Introduction

Electrical stimulation (ES) is a kind of modern treatment method, such as electroconvulsive therapy (Sackeim et al., 2000), transcranial magnetic stimulation (TMS), deep-brain stimulation (DBS), vagal nerve stimulation (Smith et al., 2005), epidural stimulation (Jahanshahi et al., 2013), and transcranial direct current stimulation (tDCS). Not only in preclinical but also in clinical studies, ES is widely proposed for use in many neurological and psychiatric disorders. However, the underlying therapeutic mechanisms remain greatly uninvestigated. Research into relation between ES and specific disorders suggests that functional recovery is attributed to following mechanisms, by means of alterations of cortical excitability (Ludemann-Podubecka et al., 2014), modulation of brain inflammatory response (Pienaar et al., 2015), blood–brain barrier permeability (Levi et al., 2012), brain perfusion and neuronal apoptosis (Borsody et al., 2014; Wang et al., 2014), and promotion of neural plasticity (Boggio et al., 2011). Neural plasticity mainly includes synapse formation, dendritic structure, and neurogenesis (Lendvai et al., 2000; Nithianantharajah and Hannan, 2006).

Neural stem cells (NSCs) are self-renewing and multipotent cells that can give rise to neurons, astrocytes, and oligodendrocytes. They exist in the subventricular zone (SVZ) of the lateral ventricle and the dentate gyrus subgranular zone (SGZ) of the hippocampus throughout life (Reynolds and Weiss, 1992; Alvarez-Buylla and Lim, 2004). Besides endogenous NSCs, there are also exogenous NSCs, which are derived from other stem cells like embryonic stem cells (Banda et al., 2015), mesenchymal stem cells (Chen et al., 2013) and induced pluripotent stem cells (Nizzardo et al., 2014) when they undergo neural differentiation. On the other hand, it has been reported that somatic cells, such as fibroblasts and astrocytes, have been reprogramed into induced NSCs with specific transcription factors (Ganat et al., 2006; Corti et al., 2012; Han et al., 2012; Thier et al., 2012). NSCs in the central nervous system (CNS) can be activated by various physiological and pathological stimuli, indicating that endogenous NSCs can be a potential therapy for brain tissue repair. In addition, the transplantation of exogenous NSCs has been verified feasible in a broad range of animal disease models (Tang et al., 2014; Salewski et al., 2015; Zhang et al., 2015) and even in certain clinical trials (Chen et al., 2013; Feldman et al., 2014). As they are transplanted or intrinsically activated, NSCs are capable to proliferate, migrate, adopt neural phenotypes, and finally integrate into neural circuits, leading to neural repair. Accordingly, NSC-based technology is a new yet promising approach for ailments in the CNS. In fact, the inadequate availability of endogenous NSCs limits CNS from self-repair in response to diseases or injuries. Similarly, difficulties in the application of NSCs transplants reduce their therapeutic efficacy. Besides well-known issues of immunological rejection, reliable sources and ethical pressure, limited proliferation, migration, differentiation, and viability of NSCs following transplantation are more challenging. For instance, literature has shown that transplanted NSCs survive for maximum several weeks (Jablonska et al., 2010). Einstein et al. (2006) discovered that neural precursor cells (NPCs) generate a few neuronal populations *ex vivo* and have low efficiency in astrocytes and oligodendrocytes differentiation after being grafted, with 15 and 7%, respectively. These limitations have impelled research workers to explore optimized and feasible protocols for NSC-based therapies.

Numerous studies have revealed that the ES plays a potential regenerative role in memory (Liu et al., 2015a), depression (Zhang et al., 2014), stroke (Guo et al., 2014), and spinal cord injury (SCI) (Becker et al., 2010) in rat models. These findings may deepen our understanding of cell replacement therapies following CNS insults and then drive the translation of NSC therapies combined with ES from animal experiments into the clinic settings. Thus, we will primarily focus on the use of endogenous and exogenous electrical currents in the development of NSC-based approaches.

## Endogenous Electrical Currents in the Central Nervous System

Endogenous electrical currents have been discovered in the normal and injured brains. These currents play an important role in biological functions, such as promotion of neural tube formation (Hotary and Robinson, 1990), induction of axonal regeneration (Borgens et al., 1980), and guidance of neural cell migration (Cao et al., 2013). For instance, Cao et al. (2013) detected that endogenous electrical currents (3–5 mV/mm) flow from the SVZ to olfactory bulb. Then they identified the applied electrical currents of physiological strength as directional signals for neuroblast migration *in vitro* and in brain slices. Data showed that directedness value of migration in electric field group is 2–2.5-folds higher than that in control group, which does not respond to electric currents. The directedness value was used to quantify directional migration of neuroblasts toward the cathode. Endogenous electrical currents also occur in pathological conditions like SCI or epilepsy. Epilepsy is characterized by non-synchronous brain electrical activity. The abnormal brain electrical activity not only results in recurrent seizure activity but also an increase of 163% in number of precursor populations in the adult dentate subgranular proliferative zone (Parent et al., 1997). The animals in this study undergo 6 h of pure electrical activation, but they have little or no injury in hippocampus. Thus, these authors preclude the possibility of injury-induced neurogenesis. However, it is unclear whether enhanced neurogenesis results in structure changes and recurrent seizures. From a regenerative standpoint, the results indicate that electrical currents could be engineered to provide directional attractive cues for driving NSC migration or regulating other cell behaviors.

Here come two questions. Whether exogenous electrical fields (EFs) can imitate endogenous signals? Can NSCs exhibit similar response to exogenous electrical cues?

## Exogenous Electrical Currents Mobilize NSCs/NPCs *in Vitro* Models

It is well established that exogenous EFs have a positive influence on cell migration known as galvanotaxis or electrotaxis since 1980s. More specifically, the cultured neural crest cells and embryonic cells move toward the cathode under the stimulation of electrical cues (Nuccitelli and Erickson, 1983; Stump and Robinson, 1983). Later studies have revealed that cathode-directed galvanotaxis is also applied to NSCs and NPCs. It suggests that EFs can guide NSCs to the lesion sites and then facilitates neural reconstruction. Several publications have shown that EFs direct migration of neonatal and adult mammalian NPCs/NSCs cathodally in a voltage or duration manner (Li et al., 2008; Ariza et al., 2010; Meng et al., 2011; Liu et al., 2015b). The major difference among these publications is the various signaling pathways mediating cell mobilization. NMDAR/Rac1/actin (Li et al., 2008), PI3K/Akt (Meng et al., 2011), and Wnt/GSK3β (Liu et al., 2015b) are involved in the complex processes, indicating that the action behind galvanotaxis is so complicated that investigators only find the tip of the iceberg.

The differentiation of cultivated NPCs/NSCs into neurons is also being amplified with exposure to ES (Li et al., 2008; Ariza et al., 2010; Feng et al., 2012; Kobelt et al., 2014). Li et al. (2008) first testified that 68% of the migrating NPCs generate immature neurons under the influence of EFs. A recent report revealed that EFs boost more mature neuronal differentiation with the help of EFs and biochemical mediums like interferon-γ (IFN-γ) (Kobelt et al., 2014). IFN-γ is a neuronal maturation factor, and the differentiation rate of NSCs is enhanced by ES. This gives hints that chemical means can be applied to NSC mobilization.

Electrical stimulation facilitates NSCs/NPCs to migrate directionally and differentiate into neurons *ex vivo*, which paves the way to regenerative medicine for CNS disorders *in vivo*.

## Exogenous Electrical Currents Mobilize NSCs/NPCs *in Vivo* Models

Although the aforementioned findings have revealed that electrotaxis and electricity-stimulated differentiation exist *in vitro*, they are insufficient to illustrate the actual effects of ES on NSCs/NPCs in CNS regeneration. For example, when NSCs/NPCs are in a more complex microenvironment *in vivo*, astrocytes become activated, resume proliferating, and form glia scar, which restrict neural regeneration (Yiu and He, 2006) following injuries. If possible, results should be confirmed in the further animal experiments and even human trials.

### Effects of Invasive Electrical Stimulation on NScs/NPcs

#### Deep-Brain Stimulation

Invasive ES always involves the usage of an electrode implantation into the brain or neuromuscle. DBS, one of the most widely investigated invasive ES, is applied in many neurological disorders, as Parkinson’s disease (PD), depression. However, the neurobiological mechanisms remain largely elusive. Some researchers propose that it potentially increases hippocampal neurogenesis. Preclinical data (Table 1) have already shown that rodents suffering from dementia, stroke, and depression have significant behavioral amelioration by DBS-induced promotion of neurogenesis (Morimoto et al., 2011; Stone et al., 2011; Schmuckermair et al., 2013; Jeong et al., 2014; Liu et al., 2015a). For example, some authors reported that improvements of cognitive function are facilitated by the stimulation of medial septum (Jeong et al., 2014), ventromedial prefrontal cortex (vmPFC) (Liu et al., 2015a), and entorhinal cortex (Stone et al., 2011), and others demonstrated the stimulation of nucleus accumbens that relieves anxiolytic symptoms (Schmuckermair et al., 2013).

**Table 1:** **Overviews of recent DBS and FES studies on NSC behavior**.

Reference	Intervention	Human/animal model	Areas of stimulation
Liu et al. (2015a)	DBS	Rat model of age-related dementia	Ventromedial prefrontal cortex
Jeong et al. (2014)	DBS	Rat model of dementia	Medial septum
Vedam-Mai et al. (2014)	DBS	Parkinson’s patients	Gpi or STN or VIM
Schmuckermair et al. (2013)	DBS	Rat model of high anxiety and depression	Nucleus accumbens
Morimoto et al. (2011)	DBS	Rat model of ischemic stroke	Striatal
Stone et al. (2011)	DBS	Rats	Entorhinal cortex
Xiang et al. (2014)	FES	Rat model of cerebral infarction	Paralyzed right forelimbs
Liu et al. (2013)	FES	Rat model of stroke	Paralyzed forelimbs

	**Results**		

Liu et al. (2015a)	Upregulates neurogenesis-related genes and NPC proliferation		
Jeong et al. (2014)	Increases cholinergic activity and neurogenesis		
Vedam-Mai et al. (2014)	Increases cellular plasticity		
Schmuckermair et al. (2013)	Enhances neurogenesis		
Morimoto et al. (2011)	Facilitates neurogenesis and angiogenesis		
Stone et al. (2011)	Increases neurogenesis and spatial memory		
Xiang et al. (2014)	Increases the number of NPCs		
Liu et al. (2013)	Modulates neurogenesis		

*GPi, internal segment of the globus pallidus; STN, subthalamic nucleus; VIM, ventrointermediate nucleus of the thalamus*.

In 2015, Liu et al. (2015a) studied whether vmPFC ES improves neuroplasticity in rats with age-related memory deficits. At the molecular level, genes (NeuN, Dcx, Angpt2, and S100a4) related with neurogenesis, neuronal differentiation, and migration in the neurogenic zones are upregulated. At the cellular level, a noticeable increase of C-Fos (a marker of neuronal activity) positive cells, which are Brdu/Dcx double-labeled, are observed, suggesting that newborn cells may contribute positive effects to better memory. Co-localization of BrdU and DCX in the hippocampus represents proliferation of NPCs. Besides proliferation, Stone et al. and Schmuckermair et al. found that DBS increases the viability of newborn neurons. Schmuckermair et al. (2013) found that BrdU-positive cells are obviously more in the group when BrdU was injected before DBS than that BrdU injected during stimulation, which result from DBS increasing survival rates of cells. Stone et al. (2011) also demonstrated that DBS promotes the survival of 10-day-old neurons before stimulation in the dentate gyrus. Critically, memory improvement is neurogenesis-dependent, for the effects can be blocked by temozolamide, a known inhibitor of neurogenesis (Stone et al., 2011).

However, enhanced anxiety-related mice do not respond sensitively to selective-serotonin reuptake inhibitors, while normal anxiety-related mice do (Schmuckermair et al., 2013). Thus, the reliability of enhanced anxiety-related animal models needs verification. Since the response to ES is dependent on time, voltage, interspecies, tissue origins, and others, investigators cannot deduce analogous neural plasticity in human from animal experiments. Short-duration stimulation is involved in the basic experiments, whereas sustained stimulation is applied in clinical settings (Stone et al., 2011). Whether chronic stimulation strengthens neurogenesis remains explored. Vedam-Mai et al. enrolled a total of 12 PD-DBS tissue samples from patients with idiopathic Parkinson’s disease who received electrode placement for about 0.5–6 years before death, which ruled out the possibility of puncture impairment-elicited neurogenesis. They first discovered NPCs increased by two to six times in the SVZ of PD-DBS brains compared with normal and untreated PD ones (Vedam-Mai et al., 2014). In addition, other possible mechanisms, like modulation of network activity and synaptic inhibition, may attribute to the effects of DBS. So the authors could not conclude that symptomatic relief in PD-DBS patients is resulted from the proliferation of NSCs. Nevertheless, DBS has vital implications for endogenous repair of the impaired brain.

#### Functional Electrical Stimulation

Functional Electrical Stimulation (FES) uses electrical currents to restore the function of the paralyzed muscles caused by SCI, stroke, and other neurological diseases. The improvements of neurological deficits may be due to FES-augmented CNS regeneration (Table 1), at least to a certain extent. Xiang and his coworkers observed that FES increases the number of NPCs in the known neurogenic niches in acute stroke rats (Xiang et al., 2014). Also, FES upregulates the expression of epidermal growth factor and basic fibroblasts growth factor, which stimulate the proliferation of NSCs/NPCs. Interestingly, given that these neural factors and NPCs peak at 7 days in the FES group, the factors may have a synergic role in FES-boosted neural plasticity. Another study also indicated that FES is beneficial for protecting cortical functions partly because it supports the reorganization of neural tissue and compensates for the lost neurons in ischemic conditions (Liu et al., 2013).

#### Electroacupuncture

Different from DBS and FES, electroacupuncture (EA) does not involve electrode implantation. EA is the combination of traditional acupuncture and a small electric current to achieve functional recovery by stimulating certain acupoints. The electric current is generated by a device, which is attached to the needles. And the needles are inserted at acupoints.

Studies have revealed that EA can improve neurobehaviors in the models of stroke (Yang et al., 2005; Kim et al., 2014), Alzheimer’s disease (Li et al., 2014), and SCI (Geng et al., 2015) perhaps via an increase of neurogenesis. Neurogenesis is wildly investigated in the cerebral ischemia model. A latest publication unraveled the molecular mechanism underlying neural regeneration elicited by EA (Kim et al., 2014) after stroke. Kim et al. tested that EA enhances the proliferation of NSCs in conjunction with increased mRNA expression and protein level, such as brain-derived neurotrophic factor (BDNF) and vascular endothelial growth factor (VEGF). Thus, BDNF/VEGF signaling pathway might engage in the neurogenesis following EA. In another study, the effects of EA on attenuating the decrease of proliferating cells and differentiated neuroblasts have also been proved to be correlated with increasing BDNF levels (Chung et al., 2015). The data indicated that BDNF plays a definitive role in the downstream pathway of neurogenesis.

### Effects of Non-invasive Electrical Stimulation on NSCs/NPCs

#### Transcranial Magnetic Stimulation

Compared with invasive ES, non-invasive electrical stimulation (NIES) does not require surgical procedures and has relative fewer side effects. It utilizes electrical or electromagnetic currents to target the brain through the scalp, leading to the change of cortical excitability, neuronal metabolisms, or neurotransmitter.

Transcranial magnetic stimulation, a commonly used NIES technique, was introduced by Barker et al. (1985) almost three decades ago. Despite the fact that TMS has received the approval from Food and Drug Administration (FDA) for clinical applications since 2008, the underlying mechanism is largely puzzling. It is widely accepted that TMS augments cerebral physiology through balancing excitatory and inhibitory activity in specific brain regions. While a relevant research has examined that TMS strengthens neurogenesis in healthy rats (Ueyama et al., 2011). That is not in line with the findings of Czeh et al. (2002). It is assumed that non-optimal TMS parameters may partly explain the insignificant effects of TMS on hippocampal neurogenesis. The differences between these two studies are frequency (25 vs. 20 Hz) and total pulses (14,000 vs. 5400 pulses), suggesting that more powerful stimulation may be more suitable for NSC activation (Ueyama et al., 2011).

In the subsequent studies, TMS can promote neurogenesis under pathological conditions, accompanied by behavior improvements (Guo et al., 2014; Zhang et al., 2014). Zhang et al. demonstrated the mechanism of deep-brain magnetic stimulation, a new non-invasive way applying a modified TMS protocol. Therefore, we classified deep-brain magnetic stimulation as TMS. Zhang and his colleagues provided evidence that TMS not only boosts the number of NPCs in a rat model of stress disorders but also facilitates the dendritic development of newborn neurons, implying that newborn neurons may probably integrate into existing neural networks (Zhang et al., 2014). How does TMS influence neurogenesis? Actually, little work has explored the mechanisms of TMS on neurogenesis. Guo et al. (2014) conducted a preliminary study, which revealed that miR-25-dependent p57 participates in the upregulation of NSCs induced by TMS in rats with cerebral ischemia. Although miR-25/p57 pathway does involve in the mobilization of TMS on NSCs, it is necessary to explore the comprehensive mechanisms. So, TMS can be a potential strategy for neural regeneration.

In some animal experiments, TMS could promote neural plasticity whether such effects can also be observed in human trials that remain to be examined. A recent clinical trial has shown that there are also similar therapeutic effects on depressed patients. Different from previous animal investigations, the clinical trial conducted by Furtado et al. (2013) measures neurogenesis *by* comparing the volume of hippocampal, amygdale, and entorhinal cortex before and after TMS using magnetic resonance imaging. Increased amygdala volumes and unchanged hippocampus volumes are found in TMS responders. There is evidence that volumetric increases are associated with structural plasticity in the CNS (Joshi et al., 2015) and neurogenesis also takes place in the amygdala (Hamilton et al., 2008). However, it is not clear whether the volumetric changes are induced by TMS or psychotropic medications, in that the study did not have a control group and all the enrolled individuals took the medications. Given that volumetric assessment was conducted 3 months later to ensure mood improvements, it is still unknown whether TMS direct enhances plasticity or improved mood leads to structural alterations. So, the relationship between nerve regeneration and TMS should deserve sustained attention in future.

#### Transcranial Direct Current Stimulation

Transcranial direct current stimulation, another commonly used NIES technique, was introduced by Priori et al. (1998) and Nitsche and Paulus (2000) following TMS. This technique delivers low currents to the brain areas of interest through electrodes over the scalp and then ameliorates negative symptoms of CNS illness by altering cortical excitability. Additionally, it is indicated that the neural regeneration can be promoted by the tDCS, thereby resulting in the improvement of neurological function.

Since fewer studies on NSC activation triggered by tDCS have been published, the level of evidence is lower than that of TMS. Rueger et al. (2012) confirmed that cathodal tDCS elicits regenerative response in stimulated hemisphere in a polarity- and session-dependent manner. Data showed that the cathodal tDCS significantly expands NSC numbers by ~60% due to facilitated proliferation and migration of endogenous NSCs. In a more recent study, tDCS promotes the mobility of exogenous NSCs, which is validated by MRI and immunohistochemistry (Keuters et al., 2015). Though anodal tDCS induces an almost double increase in the migratory activity of engrafted NSCs compared with sham and cathodal stimulation group, the migration is undirected. The cell migration distance is about 1.5 mm, which is shorter than that caused by chemotactic stimulation. The authors assumed that short-range migrating capability of implanted NSCs is restrained by the surrounding microenvironment. Perhaps, the mobility of endogenous NSCs is more susceptible to galvanotactic clues. It is worth to note that previous studies have documented that ES attracts cultured NSCs/NPCs to migrate to the cathode (Li et al., 2008; Ariza et al., 2010; Meng et al., 2011; Liu et al., 2015b). It is not consistent with the findings of Keuters and his teammates. These findings need to be replicated, and more research needs to conduct to understand how tDCs affects the migration of NSCs, and unravel their underlying electrophysiological mechanisms.

## Discussion and Future Direction

Preclinical observations suggest that adult brain can compensate for some lost neurons or tissues via enhanced endogenous activated neurogenesis or NSCs grafts. NSCs are through three distinct steps, namely proliferation, migration, and differentiation, to replenish the damaged neurons or tissues. Actually, the greatest challenges concerning application of NSCs are not only long-term cell survival, but also low proliferation, differentiation, and migration rates. There is ample evidence illustrating these hurdles. Given the complexity of CNS microenvironment, though NSCs are transplanted into an ischemic rat model, they just survive robustly (about 33.4%) when transplanted at medial coordinates while few cells survive at lateral coordinates (Kelly et al., 2004). Compared with medial coordinates, lateral coordinates are closer to lesion core. Another study reported a relatively lower viability rate, from 2 to 8% (Nakagomi et al., 2009). Even though NSCs survive, they have to differentiate to functional neurons to take effect. Yet, only 0.2% newly born neurons survive at 6 weeks following ischemia insult (Arvidsson et al., 2002), as demonstrated by Arvidsson and his coworkers. So, it is necessary to improve the poor-survival rate of newborn cells before NSC-based strategy can be applied in the clinical settings. With regard to differentiation rate, it was detected that only 6% astrocytes and no neurons present in the cortex and subcortex (Zhang et al., 2001). Finally, although Kelly et al. (2004) uncovered that transplanted NSCs migrate certain distance (about 1.2 mm) from the graft toward the lesion, they did not mention migration rate. However, other data presented that only 22.7% of surviving NSCs migrate away from the transplantation region (Muraoka et al., 2006). As outlined above, researchers should introduce efficient methods to increase viability, differentiation, and migration properties of NSCs, overcoming the above mentioned obstacles.

With the prevalence of ES application in experimental studies and clinical cases, research teams raise that it can act as an alternative modulator of NSC biology. Ongoing work has shown that ES can influence cytobiology parameters, such as growth, migration, differentiation, proliferation, and even morphology of NSCs invasively or non-invasively. Apparently, non-invasive ES has superiority over invasive ones. Most invasive ES but EA always requires implantation of a medical device with the operation of a neurosurgeon, which is time-and technology-depending. It is worthwhile to note that DBS has diminished response over time to brain stimulation. Conversely, non-invasive ES is convenient for repeated operations. Additionally, tDCS and TMS are proven to be well-tolerated, inexpensive, and safe with few adverse effects. Moreover, tDCS, a portable device with simplicity of its mode of action, offers the possibility of use as a home-based treatment (Page et al., 2015). Last but not the least, EA is minimally invasive and shares safe, efficacious, and inexpensive similarities with TMS and tDCS. Under comprehensive consideration, it is postulated that EA, tDCS, and TMS have more potential to activate NSCs in CNS.

Similar to ES, biomaterial engineering in enhancing regenerative potential of CNS has also been documented. Biomaterials, such as electrically conductive substrates (polymers and nanomaterials), have gradually earned attention. NSCs cultured on nanomaterials, namely carbon nanotubes, sprout more neuritis, and have a higher percentage of neuronal differentiation than those on conventional tissue culture plates (Huang et al., 2012). In an example of polymers used as substrates, neurons obtained under presence of ES are approximately 10% higher than that in ES absence (Pires et al., 2015). No multipotent factors directing differentiation toward neuron lineage are added to culture medium. Summing up, these biomaterials exert an impact on cultivated NSC differentiation, which is innovative while is still in its infancy. More work is needed to elucidate how biomaterials drive cellular changes in the following years.

Since physical stimuli do play an instructive role in neurogenesis, do chemical stimuli facilitate regenerative capacity of CNS? Growing evidences reveal that drug-like molecules regulate NSC-related processes. The pharmacological manipulation aims at epigenetic modifications or signaling pathways, both of which determine NSC development by influencing the property of protein (Lairson et al., 2013). Different from gene-based therapy, epigenetics regulate gene expression but cause no changes in the DNA sequence, minimizing the risk of gene mutation. Molecules targeting at epigenetic modifications include histone methylation, DNA methylation (Swaminathan et al., 2014) as well as non-coding RNA. The Ramous lab recently reported a new-discovered long non-coding RNA. The Long non-coding RNA Pinky is a regulator of neuronal differentiation and cell amplification (Ramos et al., 2015). Signaling molecules on neurogenesis, like Notch, bone morphogenetic protein, JAK–STAT, P53, and others (Lairson et al., 2013), are another current research focus. Whether these molecules act on epigenetics or signaling pathways provide insights into complex regenerative processes.

Challenges must be overcome to achieve successful cell replacement in the brain. Fortunately, exogenous ES, biomaterials, chemical stimuli as well as other cues can control NSC behaviors (migration, viability, differentiation, and proliferation). The advantages of EA, tDCS, and TMS make them an optimum ES to restore CNS dysfunctions. Due to their electrically conductive nature, nanomaterials and polymers can respond to ES. So, we presume that electricity combined with biomaterials may improve its electrophysiological features, which is beneficial for electricity to reach functional areas of deep brain even when the electrodes are placed on the scalp. Moreover, biomaterials can change a hostile microenvironment to a friendly microenvironment as they are able to deliver trophic factors for NSCs or endogenous tissues (Mahoney and Anseth, 2007). Molecules manipulate neurogenesis on one hand and depolarize cell membranes to alter the endogenous currents on the other hand. So, the use of endogenous currents induced by pharmacology is another alternative besides external ES. However, it is better to understand potency, solubility, selectivity, or pharmacokinetics of molecules prior to their application (Lairson et al., 2013). ES alone or combined with these alternative methods remains an exciting field, and decoding mechanisms behind these cues may serve to boost efficiency of neural regeneration.

## Conclusion

This review has shown that ES leads to beneficial impacts on cultured NSC behaviors and animals models of depression, stroke, AD, and SCI. These impacts may be related to strengthened intrinsic neurogenesis or increased extrinsic NSC viability and neuronal differentiation. Given that the limitations of current clinical therapies prevent neurological diseases from effective tissue repair and stem cell-based approaches are still in its infancy, ES holds great promise for facilitation of stem cell therapy. And ES thus promotes functional reconstruction in the CNS. Although a library of literature has presented data on the modulation of NSCs by ES, several aspects have not yet drawn adequate attentions. First, it needs to understand how ES acts on NSCs. Second, the detailed parameters of ES should be tailored for its maximized effects according to individual patient’s conditions. In other words, for EA, the optimal acupoints, treatment frequency, intensity, and duration need to be clarified (Ho et al., 2014). Likewise, the applications of tDCS and TMS are also related to these parameters (Table 2). Finally, what else neural structures are activated by electricity during stimulation except NSCs and neurons? The effects of ES on glia cells, local circuits, and specific molecular targets deserve further investigations (Muller-Dahlhaus and Vlachos, 2013). This may be conductive to unravel the complex processes of neurogenesis at multiple levels and study the interplay between NSCs and other neural structures.

**Table 2:** **Major studies investigating the effects of TMS, tDCS, and EA on NSCs**.

Reference	Intervention	Human/animal model	Areas of stimulation	Parameters
Guo et al. (2014)	TMS	Rat model of stroke	Primary motor cortex	300 pulses, 10 Hz, 3 s 120% M
Zhang et al. (2014)	TMS	Rat model of depression		20 pulses, 200 Hz
Furtado et al. (2013)	TMS	Depressive patients	Left DLPFC	35 trains, 10 HZ, 120% M
			Bilateral DLPFC	31 trains, 1/10 Hz, 120% M
Ueyama et al. (2011)	TMS	Normal rats		4 trains, 25Hz, 10 s, 1000 pulses
Keuters et al. (2015)	tDCS	Normal rats		Cathodal/anodal, 500 μA, 15 min
Rueger et al. (2012)	tDCS	Normal rats		Cathodal/anodal, 500 μA, 15 min
Geng et al. (2015)	EA	Rat model of SCI	Dazhui and Mingmen	2 Hz, 2 V, 30 min
Chung et al. (2015)	EA	Rat model of diabetes	Zusanliand Baihui	5/20 Hz, 2–4 mA, 20 min
Kim et al. (2014)	EA	Rat model of stroke	Baihui and Dazhui	2 Hz, 2 V, 20 min
Li et al. (2014)	EA	Rat model of AD	Baihui	2/15 Hz, 1 mA, 30 min

	**Duration**		**Results**	

Guo et al. (2014)	7 days		Increases the proliferation of adult NSCs	
Zhang et al. (2014)	Varied (depend on the purpose of experiments)		Facilitates adult hippocampal neurogenesis and maturation	
Furtado et al. (2013)	30 days		Increases amygdala volume and may promotes neurogenesis	
Ueyama et al. (2011)	14 days		Increases Brdu-positive cells	
Keuters et al. (2015)	10 days		Increases migratory activity of NSCs	
Rueger et al. (2012)	5 or 10 days		Elicits NSC activation and modulates neuroinflammation	
Geng et al. (2015)	14 days (peak)		Promotes the proliferation of endogenous neural stem cells	
Chung et al. (2015)	35 days		Increases proliferating cells and differentiated neuroblasts	
Kim et al. (2014)	10 days		Increases proliferative cells and differentiated cells	
Li et al. (2014)	20 days		Increases neurogenesis	

## Author Contributions

YH, YL, JC, HZ, and ST all participated in drafting the work. Among them, YH and YL made the largest contributions. We thank Jackson Wu who is a foreign student from Southern Medical University for language editing of the manuscript.

## Conflict of Interest Statement

The authors declare that the research was conducted in the absence of any commercial or financial relationships that could be construed as a potential conflict of interest.

## Abbreviations

AD, Alzheimer’s diseases; BDNF, brain-derived neurotrophic factor; CNS, central nervous system; DBS, deep-brain stimulation; DLPFC, The dorsolateral prefrontal cortex; EA, electroacupuncture; EFs, electrical fields; ES, electrical stimulation; FDA, Food and Drug Administration; FES, Functional Electrical Stimulation; GPi, internal segment of the globus pallidus; IFN-γ, interferon-γ; NIES, non-invasive electrical stimulation; NPCs, neural precursor cells; NSC, neural stem cell; PD, Parkinson’s disease; STN, subthalamic nucleus; tDCS, transcranial direct current stimulation; TMS, transcranial magnetic stimulation; VEGF, vascular endothelial growth factor; VIM, ventrointermediate nucleus of the thalamus; vmPFC, ventromedial prefrontal cortex.
